# Hospital Teaching Status and Patients’ Outcomes After Colon Cancer Surgery

**DOI:** 10.1007/s00268-018-4580-3

**Published:** 2018-03-23

**Authors:** Julia T. van Groningen, Eric H. Eddes, Hans F. J. Fabry, Marc W. A. van Tilburg, Ernst J. van Nieuwenhoven, Yvonne Snel, Perla J. Marang-van de Mheen, Mirre E. de Noo, On behalf of the Dutch Surgical Colorectal Cancer Audit Group and the Co-operating General Hospitals

**Affiliations:** 1 Department of Surgery, Leiden University Medical Center, Albinusdreef 2, 2333 ZA Leiden, The Netherlands; 2 Department of Surgery, Deventer Hospital, Deventer, The Netherlands; 3 Department of Surgery, Bravis Hospital, Roosendaal/Bergen op Zoom, The Netherlands; 4 Department of Surgery, Hospital St Jansdal, Harderwijk, The Netherlands; 5 Department of Surgery, Gelre Hospital, Zutphen, The Netherlands; 6 Co-operating General Hospitals, Leiden, The Netherlands; 7 Department of Biomedical Data Sciences, Leiden University Medical Center, Leiden, The Netherlands

## Abstract

**Background and objectives:**

It is increasingly accepted that quality of colon cancer surgery might be secured by combining volume standards with audit implementation. However, debate remains about other structural factors also influencing this quality, such as hospital teaching status. This study evaluates short-term outcomes after colon cancer surgery of patients treated in general, teaching or academic hospitals.

**Methods:**

All patients (*n* = 23,593) registered in the Dutch Colorectal Audit undergoing colon cancer surgery between 2011 and 2014 were included. Patients were divided into groups based on teaching status of their hospital. Main outcome measures were serious complications, failure to rescue (FTR) and 30-day or in-hospital mortality. Multivariate logistic regression models on these outcome measures and with hospital teaching status as primary determinant were used, adjusted for case-mix, year of surgery and hospital volume.

**Results:**

Patients treated in teaching and academic hospitals showed higher adjusted serious complication rates, compared to patients treated in general hospitals (odds ratio 1.25 95% CI [1.11–1.39] and OR 1.23 [1.05–1.46]). However, patients treated in teaching hospitals had lower adjusted FTR rates than patients treated in general hospitals (OR 0.63 [0.44–0.89]). However, for all outcomes there was considerable between-hospitals variation within each type of teaching status.

**Conclusion:**

On average, patients treated in general hospitals had lower serious complication rates, but patients treated in teaching hospitals had more favorable FTR rates. Given the hospital variation within each hospital teaching type, it is possible to deliver excellent care regardless of the hospital teaching type.

## Introduction

Colon cancer is one of the most common malignancies in the world. It is the third leading cause of diagnosed cancer in males and the second in females. In the Netherlands, 10,646 new patients were diagnosed in 2014 [1, 2]. Surgery still remains the cornerstone of treatment; a surgical resection is the only curative treatment modality for localized colon cancer. The goal of resection is complete removal of the colonic tumor, the major vascular pedicles and the lymphatic drainage basin of the affected colonic segment.

In the Netherlands, colon cancer surgery is performed in 87 hospitals. All hospitals provide general surgical care, both elective and urgent, for patients in their catchment area. Three different ‘hospital teaching types’ are distinguished. Academic hospitals (*n* = 8) are all associated with Dutch universities and thus are responsible for education and surgical training. Also, these hospitals function as tertiary referral centers for a selected group of patients with colon cancer. These hospitals provide high-complex and lower volume care. Second, teaching hospitals (*n* = 48) are associated with one specific academic hospital in their region. These hospitals also facilitate training of surgical residents and usually are high volume hospitals, thereby increasing possibilities for residents to gain sufficient surgical experience. And third, nonteaching or general hospitals (*n* = 31) do not facilitate surgical training. The general hospitals are usually lower volume hospitals considered to provide accessible care with the possibility to refer to teaching or academic hospitals when necessary [3, 4]. These different ‘hospital teaching types’ differ in organization of the perioperative processes, personal concerned with the ward and short or long lines of communication, all factors that are described to influence outcomes in colon cancer surgery.

In 2009, members of the Association of Surgeons of the Netherlands (ASN) initiated the Dutch Surgical Colorectal Audit (DSCA). In 2017, this audit changed into the multidisciplinary Dutch ColoRectal Audit (DCRA). The DCRA is a nationwide audit and is used to monitor, evaluate and improve quality of primary colorectal cancer care. It provides periodic feedback with a nationwide benchmark to all hospitals in the Netherlands on a set of quality measures and indicators. Already in the first years after initiation of the DCRA, a decrease in variation between hospitals and overall improvement in results on several process and outcome indicators was observed [5–7].

Until recently, hospital volume was frequently considered a surrogate for quality of care and more specifically a proxy for the experience of the team with the surgical procedure and perioperative care [5, 6]. From this perspective, the ASN in 2011 introduced a minimum annual hospital volume of 50 colorectal resections, regardless of the distribution between colon and rectal surgeries [5, 7, 8]. In addition, indicators derived from the DCRA became obligatory and are nowadays used by the Dutch Health Care Inspectorate, patient organizations and insurance companies for annual monitoring and transparency. Combining minimum hospital volume standards with the implementation of this audit has gradually become accepted as an effective way to secure quality of colon cancer care [7]. However, there still remains debate about the relation between other structural hospital factors, such as ‘hospital teaching status,’ and patient outcomes after colon cancer surgery [7, 9–12].

The objective of this study was to evaluate the short-term outcomes of patients surgically treated for colon cancer in hospitals with different ‘hospital teaching status,’ using the detailed quality indicators from the DCRA.

## Methods

Data were derived from the DCRA, a nationwide audit system containing a wide range of variables concerning diagnostics, treatments and outcomes in colorectal surgery. The dataset is based on evidence-based guidelines. To adjust for case-mix factors, the audit also contains patient and tumor characteristics. Data are collected prospectively. All 87 hospitals performing colon surgery register their patients in the DCRA. The approximate completeness in 2012 was 97% based on comparison with the Netherlands Cancer Registry. Details of the dataset, regarding data collection and methodology, have been published previously [4, 5].

### Patients

No ethical approval or informed consent was required under Dutch law. For the present analysis, all patients (*n* = 27,118) registered in the DCRA undergoing surgery for primary colon cancer between January 1, 2011, and December 31, 2014, in 87 hospitals were evaluated. Minimal data requirements to consider a patient eligible for analyses were: date of surgery, 30-day or in-hospital mortality and primary location of the tumor. A total of 108 patients were excluded due to missing data on these variables, evenly divided over different hospitals and hospital types. Patients with additional resections for locally advanced tumors or metastases were also excluded (*n* = 3417) as these procedures are mostly performed in academic or specific teaching hospitals suitable for these high-complex procedures, and this could introduce treatment by indication bias. This resulted in 23,593 included patients for the present analysis.

### Hospital teaching status and hospital volume

For this study, the hospitals were divided into three groups based on their hospital teaching status: general, teaching or academic hospital. Hospital volume was defined as the mean annual number of procedures between 2011 and 2014. Hospital volume can be seen as a proxy for experience with this procedure, and it differs considerably per hospital teaching type. We used tertiles of hospital volume in the analyses to distinguish between hospitals with relatively high versus relatively low volumes. All calculations for hospital volume were performed before exclusion of patients with additional surgery, while these surgeries also add to the experience hospitals have with the procedure.

### Outcome measures

We examined the following short-term outcome measures after colon cancer surgery: serious complications, postoperative mortality and failure to rescue. The definitions of these outcome measures are displayed in Table 1 and are based on previous studies using DCRA data [5, 6].

**Table 1:** Definitions of outcome measures

Serious complications	Percentage of patients with a serious complication leading to an in-hospital stay of more than 14 days, a surgical, endoscopic or radiological reintervention, or to death
Postoperative mortality	Percentage of patients that died within 30 days after surgery or during the first hospital admission
Failure to rescue	The percentage patients with a serious complication that died in-hospital or within 30 days after surgery

### Statistical analysis

First, patients treated in different hospital teaching types were compared on baseline characteristics and differences between these variables were analyzed using Chi-square tests. Relevant case-mix factors that were considered are: age, gender, body mass index (BMI), ASA score, Charlson comorbidity index, preoperative complications, location of the tumor, urgency and TNM stage, as described elsewhere [4, 13]. Significance was considered when the *p* value was <0.05. A *p* value <0.10 was defined as a trend toward significance, but caution is required and no definite conclusions can be drawn based on trends.

Second, multivariate logistic regression analyses were used to determine whether outcome differed between patients treated in different hospital teaching types when adjusted for differences in case-mix. Risk adjustment was done for all case-mix factors, which showed significant differences in the univariate analyses, and year of surgery, to account for possible trends over time in outcomes. Thirdly, the multivariate logistic regression analyses were repeated, adjusting for case-mix, year of surgery, and tertiles of hospital volume (as a categorical variable). This was done to test whether a relatively high hospital volume in some hospital types could (partly) explain the difference in outcomes between different hospital teaching types.

To show the magnitude of hospital variation by hospital volume within each hospital teaching type, funnel plots for all outcome measures were created. In these funnel plots, each dot represents an individual hospital, with the hospital volume (in 2011–2014) plotted on the *x*-axis and case-mix adjusted percentage of the outcome measure on the y-axis. Hospitals are displayed in different colors according to their ‘hospital teaching status.’ The average percentage of the outcome for all patients is shown by the horizontal dotted line. The 95 and 99% confidence intervals are based on a Poisson distribution varying in relation to the population size of each hospital.

Statistical analyses were performed in IBM SPSS Statistics version 22.

## Results

### Patients and treatment characteristics

A total of 23,593 patients, registered by 8 academic, 48 teaching and 31 general hospitals, were included in this study. Table 2 shows that patients treated in academic hospitals were younger and more often had a high Charlson comorbidity score compared to patients in teaching and general hospitals. Patients treated in teaching hospitals were comparable to patients in general hospitals, although in general hospitals patients more often underwent surgery in an urgent setting.

**Table 2:** Patient and tumor characteristics per hospital teaching type

	General		Teaching		Academic		
Number of hospitals	31		48		8		
Median hospital volume (before exclusion of patients with additional resections)	43 (IQR 43–61)		95 (IQR 76–124)		49 (IQR 40–63)		
Number of patients	6095		16,250		1248		

	Count	%	Count	%	Count	%	*p*
*Gender*							0.367
Male	3266	53.60	8533	52.50	658	52.70	
*BMI categories*							<0.001
<18.5	78	1.30	254	1.60	30	2.40	
18.5–25	2222	36.50	6432	39.60	494	39.60	
25–30	2331	38.20	6244	38.40	482	38.60	
30+	982	16.10	2603	16.00	210	16.80	
Unknown	482	7.90	717	4.40	32	2.60	
*Age*							<0.001
≤60	955	15.70	2512	15.50	242	19.40	
61–70	1740	28.60	4819	29.70	391	31.30	
71–80	2195	36.00	5759	35.50	426	34.10	
≥81	1203	19.70	3152	19.40	189	15.10	
*Charlson score*							<0.001
Charlson score 0	2956	48.50	7969	49.00	512	41.00	
Charlson score 1	1478	24.20	3833	23.60	257	20.60	
Charlson score 2+	1661	27.30	4448	27.40	479	38.40	
*ASA score*							0.003
I–II	4453	73.40	12,092	74.50	880	70.50	
III	1485	24.50	3852	23.70	336	26.90	
IV–V	131	2.20	281	1.70	32	2.60	
*Location primary tumor*							<0.001
Cecum	1211	19.90	2983	18.40	248	19.90	
Appendix	49	0.80	84	0.50	18	1.40	
Ascending colon	1146	18.80	3052	18.80	206	16.50	
Hepatic flexure	386	6.30	1010	6.20	80	6.40	
Transverse colon	391	6.40	1201	7.40	112	9.00	
Splenic flexure	198	3.20	503	3.10	42	3.40	
Descending colon	365	6.00	972	6.00	68	5.40	
Sigmoid colon	2349	38.50	6445	39.70	474	38.00	
*Pathological T stage*							<0.001
T1 and ypT0	497	8.15	1368	8.41	163	13.06	
T2	1047	17.18	2801	17.24	212	17.00	
T3	3697	60.66	9954	61.26	737	59.05	
T4	793	13.01	1972	12.14	130	10.42	
Missing	61	1.00	73	5.85	25	2.00	
*Pathological N stage*							0.011
N0	3612	59.3	9634	59.3	767	61.50	
N1	1487	24.40	3987	24.50	291	23.30	
N2	935	15.40	2500	15.40	168	13.50	
Unknown	57	0.90	124	0.80	21	1.70	
*Metastasis (without additional surgery)*	620	10.20	1495	9.20	117	9.40	<0.001
*Preoperative tumor complication*	2709	44.40	6657	41.00	536	42.90	<0.001
*Urgent setting*	1176	19.30	2783	17.10	217	17.40	0.001
*Type of surgery*							0.001
Laparotomy	2709	44.50	7075	43.60	581	47.20	
Laparoscopy	3383	55.50	9163	56.40	651	52.80	

In addition, it is shown that hospital volume was higher in teaching hospitals compared to general and academic hospitals (Table 2). Hospital volumes of each group increased over the last 4 years. In 2011, general hospitals had a median hospital volume of 53 patients (IQR 42–62), increasing to 57 (IQR 41–69) in 2014. The same trend is observed in both teaching and academic hospitals ranging from 85 (IQR 68–112) and 44 patients (IQR 32–52), respectively, in 2011, to 111 (IQR 85–138) and 68 (IQR 38–84) patients in 2014.

### Outcomes

#### Serious complications

Table 3 shows the difference in serious complication rates of patients treated in general hospitals (15.6%) and patients treated in teaching (17.6%) and academic hospitals (18.3%). After adjusting for case-mix and year of surgery, patients treated in teaching and academic hospitals had significantly higher serious complication rates than patients treated in general hospitals, with odds ratio of 1.22 [95% CI 1.13–1.33] and 1.23 [1.05–1.45]. After also adjusting for hospital volume, the effect did not change much (OR 1.25 [1.12–1.39] and 1.23 [1.05–1.46]), indicating that volume does not explain this difference in serious complication rates. Teaching and academic hospitals showed no significant difference (data not shown).

**Table 3:** Unadjusted percentage and multivariate regressions of the outcome measures: serious complications, postoperative mortality and failure to rescue

Outcome	Unadjusted percentage (*n*/*N*)	Multivariate regression**			Multivariate regression (incl. volume)***		
		Odds	95% C.I. for EXP (B)		Odds	95% C.I. for EXP (B)	
			Lower	Upper		Lower	Upper
**Serious complications**			c-stat: 0.649			c-stat: 0.651	
General (ref.)	15.6% (948/6095)	1			1		
Teaching	17.6% (2856/16,250)	**1.22**	**1.13**	**1.33**	**1.25**	**1.12**	**1.39**
Academic	18.3% (228/1248)	**1.23**	**1.05**	**1.45**	**1.23**	**1.05**	**1.46**
**Postoperative mortality**			c-stat: 0.819			c-stat: 0.819	
General (ref.)	3.3% (201/6095)	1			1		
Teaching	3.0% (492/16,250)	1.04	0.87	1.24	1.05	0.84	1.33
Academic	3.4% (41/1248)	1.14	0.80	1.63	1.14	0.80	1.63
Failure to rescue*			c-stat: 0.748			c-stat: 0.750	
General (ref.)	18.3% (119/649)	1			1		
Teaching	14.7% (304/2073)	0.78	0.60	1.00	**0.62**	**0.44**	**0.88**
Academic	7.9% (23/164)	0.77	0.46	1.29	0.74	0.44	1.24

Bold values are the significant differences, with a confidence interval that does not cross 1

*Denominator: patients with a serious complication that underwent elective surgery

**Adjusted for: case-mix and year of surgery

***Adjusted for: case-mix, year of surgery and hospital volume in tertiles

**Fig. 1:**
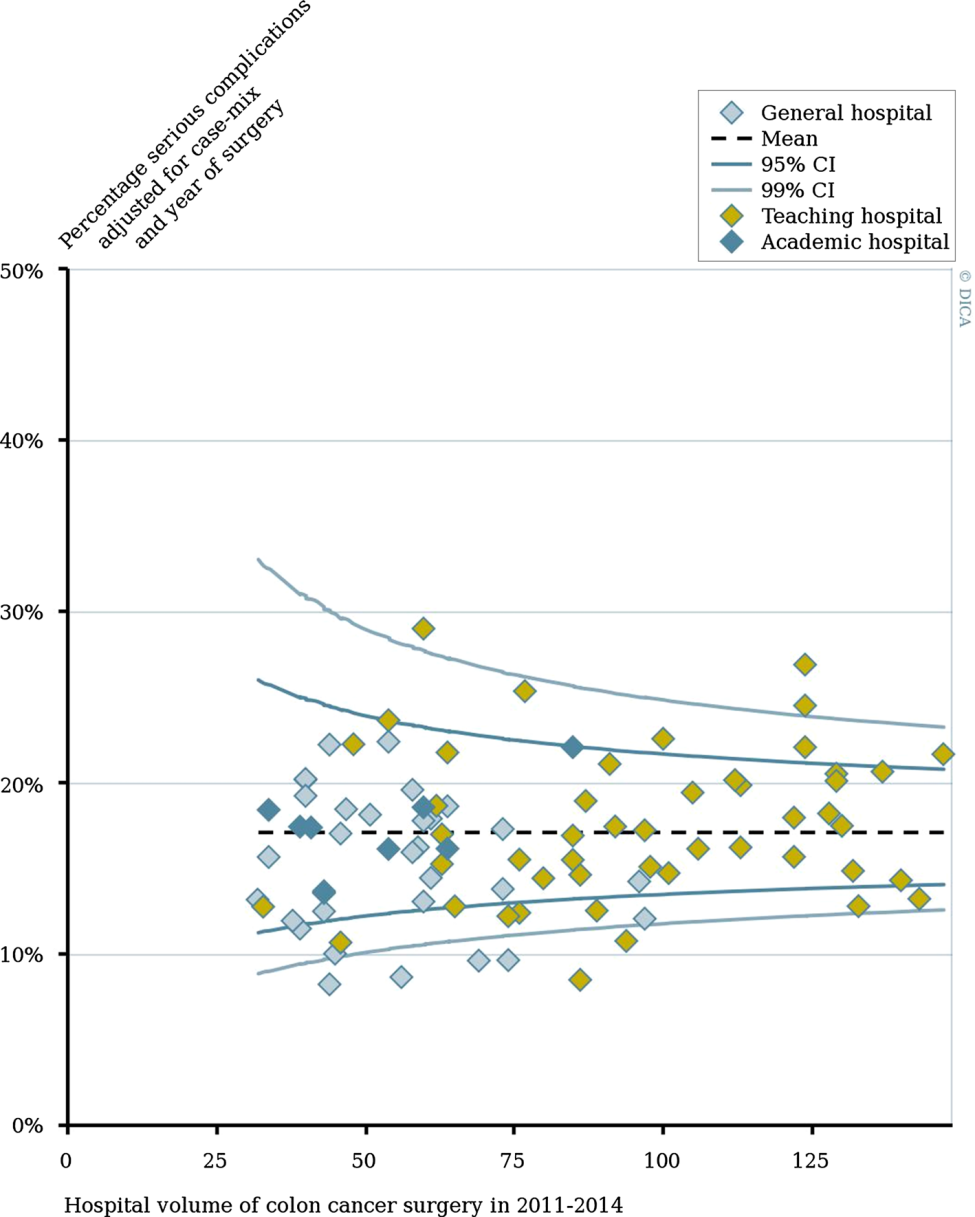
Percentage of serious complications after colon cancer surgery according to hospital type and hospital volume in 2011–2014. Percentage of each hospital was adjusted for case-mix and year of surgery

Figure 1 shows the variation in adjusted serious complication rates among the Dutch hospitals. The funnel plot demonstrates serious complication rates and hospital volume within general, teaching and academic hospitals. It demonstrates that within each category of teaching status, variation in hospital volume and risk of complications was observed. In addition, all negative outliers were teaching hospitals, while positive outliers were general and teaching hospitals. It also showed that the results from of the regression model are not due to these few outliers.

#### Failure to rescue

Patients treated in teaching hospitals showed a trend toward lower failure to rescue rates as compared to general hospitals after adjusting for case-mix and year of surgery; however, this effect was not significant (Table 3). After additional adjustment for hospital volume, the failure to rescue rate of patients treated in teaching hospitals compared to general hospitals was significantly lower (OR 0.63 [0.44–0.89]). Academic hospitals showed an effect in the same direction, but this was not significant. Teaching and academic hospitals showed no significant difference (data not shown).

**Fig. 2:**
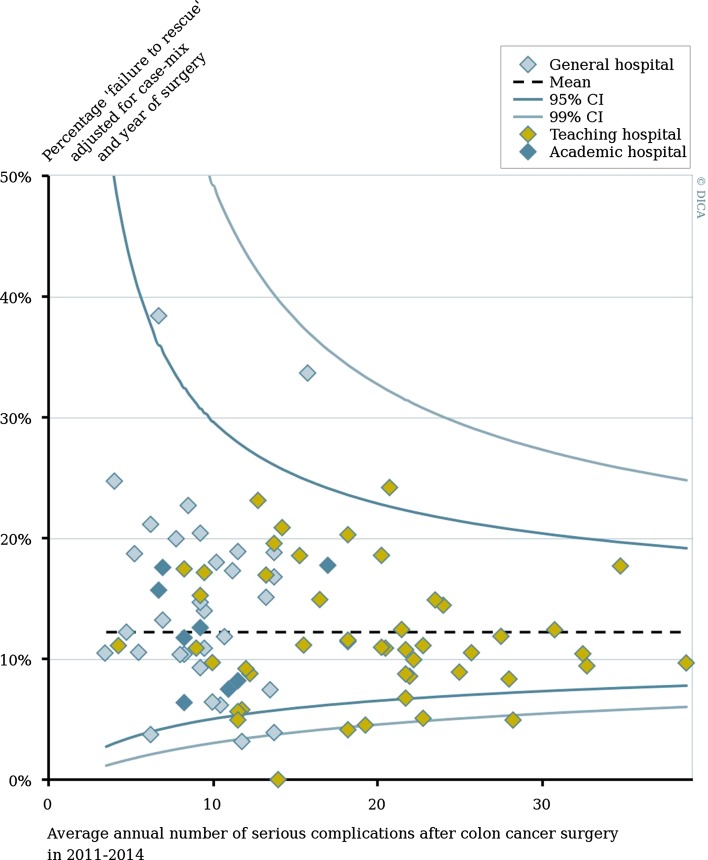
Percentage of failure to rescue after colon cancer surgery according to hospital type and hospital volume of serious complications in 2011–2014. Percentage of each hospital was adjusted for case-mix and year of surgery

Figure 2 shows the considerable variation between hospitals in failure to rescue rates within different hospital teaching types. Nine hospitals had significantly lower failure to rescue rates than the Dutch average of 12.2%; both general and teaching hospitals were represented in these positive outliers. Two general hospitals and one teaching hospital had a significantly higher failure to rescue rate than the Dutch average.

#### Postoperative mortality

Patients treated in different hospital types showed no difference in 30-day or in-hospital postoperative mortality after colon cancer surgery (Table 3). Variation in postoperative mortality between hospitals showed a similar pattern to that of failure to rescue (data not shown).

## Discussion

The present study showed that patients treated in teaching hospitals and academic hospitals on average had higher serious complication rates than patients treated in general hospitals after primary colon cancer surgery when adjusted for case-mix and year of surgery. Hospital volume could not explain these differences. However, patients treated in teaching hospitals had lower adjusted failure to rescue rates than patients treated in general hospitals. Postoperative mortality did not differ between patients treated in different hospital types. For all these outcomes, considerable hospital variation was shown within all hospital teaching types and hospitals with best outcomes are found in all hospital teaching types.

The results of this study contribute to the discussion whether certain structural factors, such as teaching status, are related to good outcomes after colon cancer surgery. Previous studies have suggested that hospital teaching status is associated with outcomes for patients treated for several medical conditions and after several surgical procedures. In most studies, outcomes were favorable for patients treated in teaching and academic hospitals [6, 10, 14–18]. However, (the size of) the association between teaching status or hospital volume and postoperative mortality seems to differ considerably per procedure and condition [6, 8, 10, 14–21]. In colorectal cancer surgery, previous studies in the Netherlands and Canada showed no or a small effect of hospital teaching status on postoperative mortality of patients [21, 22]. However, Friese et al. [20] showed no effect of hospital teaching status on failure to rescue and postoperative mortality in surgical oncology, after risk adjustment. Furthermore, Elferink et al. found that patients treated in general hospitals had lower odds on ≥10 lymph nodes adequately investigated and higher odds for receiving adjuvant therapy. The postoperative mortality did not differ with different structural factors. However, survival was suggested to be better for patients treated in university hospitals [11].

The present study adds to this literature by showing the association on several short-term outcome indicators. It confirms the absence of an association between teaching status and postoperative mortality after colon cancer surgery, but adds that patients treated in different hospital types on average seem to differ in risk of serious complication and failure to rescue. However, we also showed considerable hospital variation within each hospital teaching status. Part of this hospital variation in outcomes might be explained by differences in process indicators as shown by Elferink et al. [11] within each teaching group. This might also explain that best performers are found in all hospital teaching types, suggesting that all hospitals can achieve good outcomes after colon cancer surgery, regardless of their difference in hospital teaching status (and hospital volume).

The question remains whether it is the effect of the hospital teaching status on short-term outcomes or rather a combination of processes more prevalent in certain hospital types that lead to better outcomes. If it is indeed this combination of processes, identifying these processes might be more effective than using indirect factors that are not amenable for change. Suggested factors accounting for variation are for instance: difference in advanced technology and nurse staffing [6, 7, 9, 11]. Possible explanations could also be surgeon specific, such as degree of specialization, availability during on-call hours and efficient escalation of care from nurses to specialized surgeons. All of these factors might contribute to preventing that a patient develops a serious complication or even death [3, 23–25]. Further research, measuring these potentially explanatory factors, might be relevant to improve quality of all hospitals, independent of their hospital teaching type.

Some limitations should be noted. First, selection bias cannot be completely excluded, as doctors report the data themselves. However, as is shown in previous publications, the dataset is detailed and frequently validated both internal and against other external sources [4–6, 12, 13]. Furthermore, although we adjusted for a variety of most relevant case-mix factors, unknown confounding case-mix factors may possibly play a role such as medication or smoking habits [4, 13]. Last, we used data derived from the Dutch population so it is not known if our results can be generalized to other countries. Factors that may influence this generalization are: the minimum volume standard that is in place, whether a clinical audit system is performed, but also density of the population and thus access to hospitals and distribution of hospitals over the country.

So, even though we have shown that on patient-level hospital teaching status was significantly related to lower risk of serious complication in general hospitals and lower failure to rescue rates in teaching hospitals, considerable between-hospitals variation was shown regardless of teaching status. Best performers are found in all hospital teaching types, which suggests that is it possible to deliver excellent care in each hospital teaching type. This study shows that to learn from best performers further research should start looking for other factors than structural factors to improve our outcomes in colon cancer surgery.
